# Reproduction of Sheep through Nuclear Transfer of Somatic Cells: A Bibliometric Approach

**DOI:** 10.3390/ani13111839

**Published:** 2023-06-01

**Authors:** José Roberto Vazquez-Avendaño, César Cortez-Romero, Ángel Bravo-Vinaja, Demetrio Alonso Ambríz-García, Alfredo Trejo-Córdova, María del Carmen Navarro-Maldonado

**Affiliations:** 1 Doctorado en Ciencias Biológicas y de la Salud, Universidad Autónoma Metropolitana, Ciudad de México C.P. 3855, Mexico; robertmizer@gmail.com; 2 Department of Biology of Reproduction, Division of Biological and Health Sciences, Universidad Autónoma Metropolitana, Unidad Iztapalapa, Ciudad de México C.P. 09310, Mexico; deme@xanum.uam.mx (D.A.A.-G.); atrejo109@hotmail.com (A.T.-C.); 3 Program in Genetic Resources and Productivity-Livestock, Campus Montecillo, Colegio de Postgraduados, Montecillo, Texcoco C.P. 56264, Mexico; ccortez@colpos.mx; 4 Program in Innovation in Natural Resources Management, Campus San Luis Potosí, Colegio de Postgraduados, Salinas de Hidalgo, San Luis Potosí C.P. 78600, Mexico; abravo@colpos.mx

**Keywords:** SCNT, nuclear transfer, reproductive biotechnology, bibliometric analysis, sheep, VOSviewer

## Abstract

**Simple Summary:**

Different reproductive biotechnologies have been applied to sheep, such as cloning, which has been successfully applied in this species. In this context, the aim of the present study was to carry out a bibliometric analysis of the scientific literature on cloning applied to sheep reproduction, since the first report was published, to identify the most cited articles, main authors and collaboration among them, published journals, institutions with more published papers, most prolific countries and the network collaboration among them, and research topics. This study collected bibliographic data from 124 papers relating to cloning of sheep. The articles that were cited more often addressed topics related to the generation of transgenic animals, recovery of wild species, and xenotransplants. So far, no bibliometric studies have been conducted about cloning of sheep.

**Abstract:**

Somatic cell nuclear transfer (SCNT) is a reproductive biotechnology with great potential in the reproduction of different species of zootechnical interest, including sheep. This study aimed to carry out a bibliometric analysis of scientific papers published on the application of SCNT in sheep reproduction during the period 1997–2023. The search involved the Science Citation Index Expanded and Social Sciences Citation Index databases of the main collection of the Web of Sciences with different descriptors. A total of 124 scientific papers were analyzed for different bibliometric indicators using the VOSviewer software. Since 2001, the number of SCNT-related papers that have been published concerning sheep reproduction has increased and it has fluctuated in ensuing years. The main authors, research groups, institutions, countries, papers, and journals with the highest number of papers related to the application of SCNT in sheep reproduction were identified, as well as the topics that address the research papers according to the terms: somatic cell, embryo, oocyte, gene expression, SCNT, and sheep.

## 1. Introduction

Sheep are one of the first species to have been domesticated from about 8000 to 9000 years ago. The adaptability of this species to different types of weather has allowed their wide geographic distribution. The global population of sheep reached 1.2 billion by the year 2012. Sheep are among the top five most economically important domestic species in the world, and approximately 1400 discrete breeds have been registered [1]. Sheep are easy to manage and maintain, and their pregnancy period is relatively short, which are advantageous for evaluating genetic improvement programs [2,3].

There are different reproductive biotechnologies that have focused on sheep which include artificial insemination (AI), in vitro fertilization (IVF), gamete and embryo cryopreservation, cloning, among others [4]. In vitro production of cloned embryos involves vertical and horizontal approaches [5]. The vertical approach includes the generation of monozygotic twins from blastomere separation and embryo bipartition in vivo or by IVF [6]. In contrast, the horizontal approach involves somatic cell nuclear transfer (SCNT). In SCNT, an oocyte devoid of its nucleus serves as a cytoplasmic receptor for donated genetic information from a somatic cell [7]. The first attempts to generate cloned embryos by blastomere separation and embryo bipartition were carried out in sheep, as was the generation of embryos by SCNT [8]. SCNT is a biotechnology with great potential to reproduce sheep with high genetic value [9], to conserve endangered wild sheep [10], and to generate transgenic sheep with biomedical purposes [11].

The scientific and technological advances in the past several decades have been reflected in an increase in scientific information in bibliographic databases for the dissemination of knowledge. This has enhanced the use of bibliometrics [12]. Bibliometrics is a method to help evaluate scientific information by evaluating a set of methodological knowledge in published papers through indicators, number of papers published, and citations of these papers, according to the region or country of origin, authors, working groups, and research centers [13]. Bibliometric studies have been used to quantify scientific output and to identify groups and areas of excellence, thematic and interdisciplinary emerging disciplines, and thematic collaboration networks [12]. Governments can use this information to implement policies that benefit the scientific and technological development of their nations [14]. 

An evaluation of the different elements of scientific papers can reveal different bibliometric indicators that measure the results of scientific and technological work. The choice of the database to be used in the analysis of scientific information will condition the bibliometric indicators that can be developed [12]. 

The aim of this study was to identify regularities of scientific information to provide an overview of scientific research published in mainstream journals on the application of SCNT in sheep reproduction. The study used several one-dimensional and multi-dimensional bibliometric indicators. The data were analyzed using the VOSviewer software. 

## 2. Materials and Methods

The Science Citation Index Expanded (SCIE) and the Social Sciences Citation Index (SSCI) databases of the Web of Science were used to search for papers related to the application of SCNT in sheep reproduction that had been indexed in these databases and published in mainstream journals [15] from 1997 to 2023. The expressions used in the advanced search option in the search performed in January 2023 were TS = (“Somatic cell*” “Nuclear transfer”) OR SCNT) and TI = (Ewe OR Sheep OR Ovine OR “Lamb*”). These words were searched for in the titles, abstracts, and keywords of the scientific papers. Only research and review papers were considered. The bibliographic records that were obtained were analyzed according to the one-dimensional and multi-dimensional indicators [16,17] shown in Table 1.

The bibliographic data were analyzed using Excel to obtain the “literature growth” indicator. Data obtained from the other indicators were analyzed using the VOSviewer software version 1.6.19 (Centre for Science and Technology Studies, Leiden University, Leiden, The Netherlands, 2023), which visualizes scientific landscapes. This bibliometric software was used, in the present study, to create and visualize maps by “visualization of similarities” (VOS), a method proposed by van Eck and Waltman [18]. This is an alternative to multi-dimensional scaling to visualize similarities between themes or objects. A co-occurrence analysis of the words related to sheep SCNT was performed in the titles and abstracts of the scientific papers [19]. The generated maps of science featured scientific thematic networks between teams of researchers, institutions, and countries concerning the application of SCNT in sheep. The terms were standardized before analysis. A joint word analysis was also performed, which was extracted from the titles and abstracts of the published papers. This co-occurrence of words reflects the conceptual relationship network of the views of scientists active in the field. The frequency of words was used to construct co-occurrence maps representing the intellectual content of an area of research through the analysis of groups and networks [20].

## 3. Results

### 3.1. Growth of Literature

**Figure 1:**
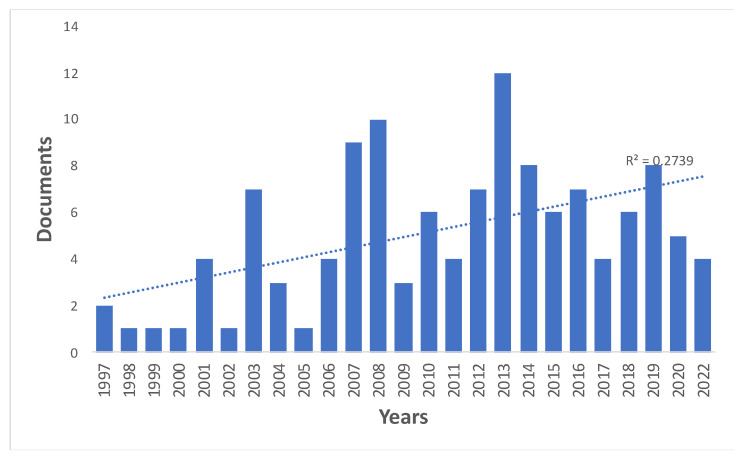
Distribution of papers about the application of SCNT in sheep, indexed in Web of Science from 1997 to 2022.

The Science Web reference and citation database revealed 107 papers. In addition, 17 papers were included from the *Journal Citation Reports* (JCR) that had not been identified in the initial search. In total, there were 124 papers that comprised 118 research papers (95.2%) and 6 review papers (4.8%). The first paper on the application of SCNT in sheep, entitled “Viable offspring derived from fetal and adult mammalian cells”, was published in 1997 in the journal *Nature* [21]. In subsequent years from 2001, the number of published papers fluctuated from one to twelve papers every year, with an annual average of five papers. Most papers (*n* = 12) were published in 2013 (Figure 1).

### 3.2. Most Productive Author

Keith Henry Stockman Campbell published the most papers about SCNT in sheep. The 15 articles published have been cited 4868 times (H-index of 34) [22]. Dr. Campbell was affiliated with the University of Nottingham until his death in 2012. One aspect of his research was the application of SCNT in mammals, using sheep as a research model for the generation of cloned or transgenic lambs. The author with the highest scientific productivity is Professor Sir Ian Wilmut, who has published nine articles that have been cited 4992 times (H-index of 69). His research at the University of Edinburgh focused on cell reprogramming mechanisms and regenerative medicine (Table 2).

### 3.3. Cooperation between Authors

**Figure 2:**
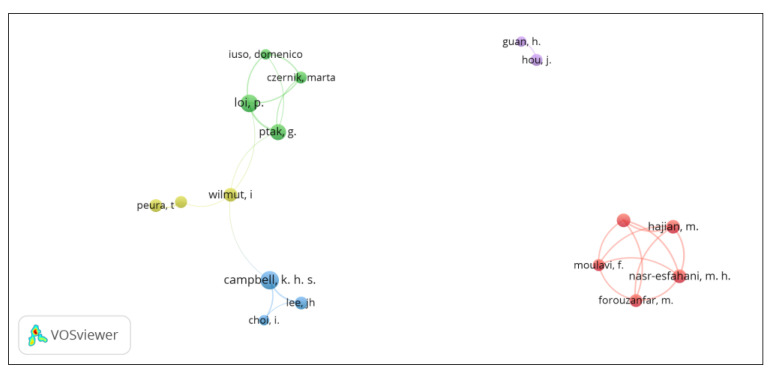
Visualization of research groups on the application of SCNT in sheep using the VOSviewer software.

There are five research groups that focused on the application of SCNT in sheep (Figure 2). The group with the largest number of researchers is composed of Samaneh Sadat Hosseini Farahabadi, Mehdi Hajian, Mohammad Hossein Nasr-Esfahani, Forouzafar Mohsen, and Fariba Moulavi. They belong to different institutions in Iran, including the Royan Institute for Biotechnology, the Islamic University of Azad, and the Camels Advanced Reproductive Technologies Center. This research group did not collaborate with others. The second research group is composed of four researchers: Pasqualino Loi, Marta Czernik, and Domenico Iuso from the University of Teramo in Italy and Grażyna Ptak from the Jagellonian University in Poland. The third research group includes the aforementioned Dr. Campbell, Joon Hee Lee, and Inchul Choi from the University of Nottingham in England. The fourth research group includes Teija Peura of Genea Biomedx (Box Hill, Australia), Simon Walker of the Turretfield Research Centre, (Rosedale, Australia), and the aforementioned Professor Sir Wilmut. Research groups from two to four collaborated with each other. The final research group includes Jian Hou and Hong Guan of the Chinese University of Agriculture, who have had no interaction with other research groups.

### 3.4. Published Papers and Cooperation among Countries

**Figure 3:**
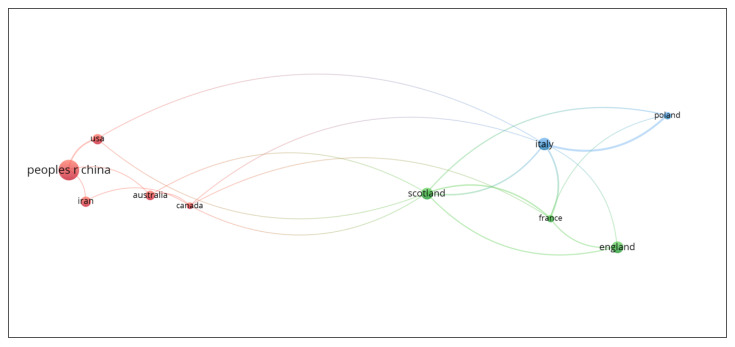
Visualization of countries with the greatest contribution of published papers about the application of SCNT in sheep and their collaborative relationships using the VOSviewer software.

The countries that have most intensively performed and published research on SCNT involving sheep are shown in Table 3. The collaboration among countries is shown in Figure 3. Scholars from China have collaborated mainly with colleagues from Iran, Australia, Canada, and United States. Scholars from England have collaborated with colleagues from Scotland and France. Finally, academics from Italy have collaborated with colleagues from Poland.

### 3.5. Institutions with the Largest Contribution of Published Papers

The University of Nottingham has the highest number of published papers concerning the application of SCNT in sheep, with 14 papers. In accordance with the Academic Ranking of World Universities (ARWU) and the World Classification of Universities (QS), it is ranked 101–150th and 114th, respectively. The Roslin Institute of the University of Edinburgh holds the second position, with 13 published papers that have been cited 1844 times, seven papers have been cited more than 100 times each. The University of Edinburgh occupies position 35th in the ARWU and position 15 in the QS (Table 4).

### 3.6. Journals

The eight main journals with papers dealing with SCNT-related topics in sheep are shown in Table 5. The information in the table includes bibliometric data and impact factor (IF) [23]. It also includes the positions of the journals in accordance with the thematic category, which indicates the quartile according to the JCR. IF is used as an indicator of the relative importance of a journal within a particular area of study and it evaluates the frequency with which journal articles are cited during a given period [24]. The journal *Cellular Reprogramming* has published the most papers related to SCNT in sheep (*n* = 11, IF 2.257, JCR position in the fourth quartile of thematic categories). The next journal is *Theriogenology* (*n* = 10, IF 2.923, first quartile for the “Veterinary Sciences” category). The journal with the highest number of citations is *Biology of Reproduction* (second quartile of the “Reproductive Biology” category), followed by *Theriogenology* (first quartile in the “Veterinary Sciences” category).

### 3.7. Most Cited Articles

The citation structure of papers between 1997 and 2022 included five papers with ≥180 citations (Table 6). The most cited article referred to the first lamb cloned from adult cells obtained from mammary gland; this success demonstrated two facts, i.e., first, terminally differentiated cells preserve all their genetic information, and it can be restored; and second, the SCNT can be applied to superior vertebrates such as mammals [21].

The second most cited article focused on the generation of transgenic lambs from the transfection of fetal fibroblasts with the neomycin resistance marker gene and human coagulation factor IX, for protein coding in sheep’s milk [25]. This was followed by an article related to the generation of transgenic lambs. It focused on “gene targeting” in fetal fibroblasts to integrate a therapeutic transgene into the gene locus ((COL1A1) collagen type I, alpha 1 chain) in sheep [26]. The fourth most cited article was related to SCNT as a tool for the recovery of wild species at risk, in which the feasibility of using *Ovis aries* oocytes as receptors of *O. orientalis musimon* fibroblasts for interspecific SCNT. One of the embryos reached the term of gestation with the birth of a European mouflon [10]. 

Five articles were cited from 100 to 179 times. For example, Beaujean et al. [27] evaluated the dynamics of somatic nucleus methylation after SCNT at different stages of early embryonic development, showing that the demethylation process failed in the trophectoderm cells of blastocysts. 

Six other articles were cited from 50 to 99 times. One study [32] evaluated the imprinting status of insulin-like growth factor 2 and H19 gene (IGF2-H19) and insulin-like growth factor 2 receptor (IGF2R) in lambs generated by SCNT. The authors observed that one lamb displayed deregulation in the imprint of the second loci intron of the IGF2R gene. 

Three articles were cited from 40 to 49 times. One study [37] evaluated the expression of mitochondrial DNA replication factors encoded by the nucleus and expressed for the first time in the later stages of early embryonic development. 

The nine articles with from 30 to 39 citations include Peura and Vajta [39] who described a new SCNT method for cattle and sheep (handmade cloning), characterized by using pelucide-zone-free oocytes and the absence of micromanipulators to enucleate them. 

Seven articles were cited from 20 to 29 times. They included Zhang et al. [11] who described generating transgenic lambs with high levels of omega-3 fatty acids using handmade cloning. The authors concluded that handmade cloning efficiency was similar to the conventional technique for the generation of transgenic animals. 

Twenty-six articles were cited between 19 and 10 times. Among them, Wen et al. [40] tested two inhibitors of histone deacetylases, trichostatin A, and scriptaid. The latter improved the epigenetic status of ovine embryos via SCNT. 

Finally, 52 articles were cited ≤9 times. Choi et al. [41] combined calcium ionophore, strontium chloride, and cytochalasin B to activate cario-cytoplast complexes, improving embryo quality. Among these articles, 10 articles had no record of citations. The most recent article was by McLean et al. [42] who studied the effect of embryo aggregation during the vitrification process of cloned sheep embryos. They observed that the aggregated embryos had an in vivo survival rate similar to that of the group that was not vitrified. The oldest most cited published paper is that of Wilmut et al. [21]. Table 6 includes the articles with the highest number of citations between 1997–2022.

### 3.8. Identification of Research Topics

The results of the co-occurrence analysis revealed 553 words or terms that were used most frequently in scientific papers. Only those with more than five co-occurrences were considered. The resulting 52 terms were organized into five clusters. The 14 words or terms most frequently used in the published papers are listed in Table 7. This analysis clarified the main topics of interest in the study area.

**Figure 4:**
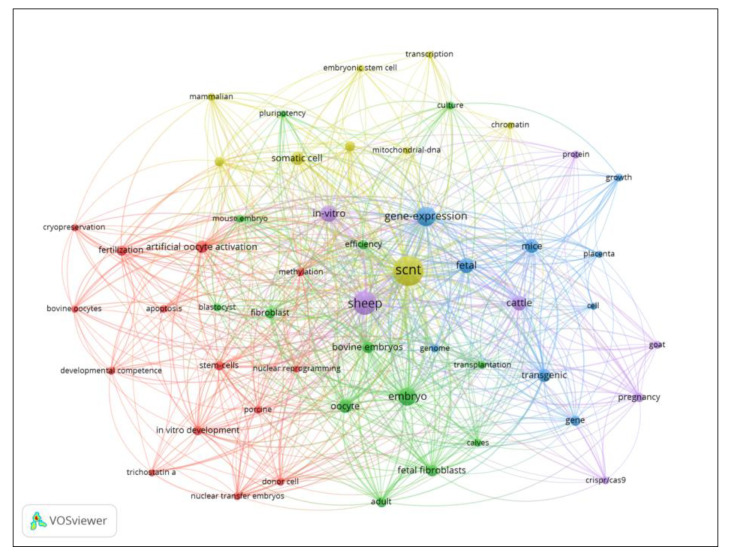
Network map of themes about the application of SCNT in sheep, grouped in clusters.

Figure 4 displays five clusters of words (a cluster is a group of words related to each other) and the relationships among the clusters. Each cluster is represented by one color, cluster one has 14 words (red), cluster two includes 13 words (bright green), cluster three involves 9 words (blue), cluster four contains 9 words (light green), and cluster five has five words (purple).

## 4. Discussion

In the present study, 124 papers on the reproduction of sheep by applying SCNT were counted in the WOS database; this quantity of papers was similar to that reported in a review carried out for the 25th anniversary of cloning using SCNT. This review published a survey of all published papers (1997–2020) on SCNT classified by species, among which mice, cattle, pigs, goats, and sheep were the species for which more than 100 published papers had been registered [43].

Since the publication of the study on the birth of the Dolly sheep (1997), the number of papers published per year has fluctuated, with an average of five papers per year. However, a more detailed analysis shows that, during the first (1997–2006) and second (2007–2016) decades, the numbers of published papers were 25 and 72, respectively, while in the last period (2017–2022), 27 papers were published, indicating a general growth in research on the reproduction of sheep by cloning.

China, Italy, England, and Scotland are the countries that have generated more than 50% of the scientific research on the reproduction of sheep using SCNT. China is the world’s leading producer of sheep, while Australia, Iran, and the United Kingdom (which includes England and Scotland) are among the ten countries with the highest number of sheep worldwide, according to FAO data for 2020 [44]. This could partly explain the interest of these countries in research on the reproduction of sheep by cloning.

The universities that have shown interest in the reproduction of sheep by cloning, are the University of Nottingham (England), the Roslin Institute of the University of Edinburgh (Scotland), the University of Teramo (Italy), and the China Agricultural University (China), which are the affiliations from the most cited authors in this area of research.

The principal journals that have published topics related to sheep reproduction by applying SCNT are *Cellular Reprogramming*; *Theriogenology; Reproduction, Fertility and Development*; *Molecular Reproduction and Development*; *Plos ONE*; and *Reproduction in Domestic Animals*. These journals have published 37.9% of all published papers. The most frequent JCR categories in these journals are “reproductive biology”, “veterinary sciences”, “developmental biology”, and “agricultural dairy animal sciences”, and therefore, these journals are the most suitable for consulting or publishing on this area of research.

The most cited article reports the birth of Dolly [21], obtained from a somatic cell from the mammary gland of an adult sheep, an unusual fact that had not been achieved in an upper mammal and which was a watershed for science, and therefore, for this reason, this article is still widely cited today. The other two most cited articles address issues about the generation of transgenic lambs for the production of human proteins for therapeutic purposes, which means that cell lines expressing a specific gene in the SCNT can be used, and this ensures that a lamb is obtained with the desired modification [25,26].

The words “oocyte” and “somatic cell” were frequent, and both cell types were used for SCNT. Oocytes have been treated with caffeine, which increases the activity of the promoter factor of maturation and of mitogen-activated protein kinases that are important for the nuclear reprogramming process during SCNT embryo development [33,45,46]. Moreover, the words “embryo”, “gene expression”, and “in vitro” were used in connection with each other. Examples include studies that have evaluated the effect of different chemical agents on the state of DNA methylation [47,48] and inhibitors of histone acetyltransferases [40,49] in the development of in vitro cloned embryos, especially at the blastocyst stage.

A detailed analysis of Figure 4 shows that cluster one, in red, contains 14 words related to the competence of in vitro cloned embryos. Khan et al. [50] compared the efficiency of conventional SCNT and handmade cloning for generating cloned sheep embryos. Better rates of efficiency of enucleation and fusion were obtained with handmade cloning, as well as a higher percentage of segmented and blastocyst stage embryos.

Cluster two, in bright green, contains 13 words referring to the development efficiency of cloned embryos at the blastocyst stage using different types of cells as donors of genetic information, most commonly cumulus cells and fibroblasts. The cells were treated with egg extracts [51], zebularine [47], chaetocin [52], and histone demethylase enzymes [53] to promote nuclear reprogramming of somatic cells and to improve the epigenetic status of ovine cloned embryos. 

Cluster three, in blue, contains nine words focusing on the fetal development of cloned sheep and their regulation of gene expression. Ni et al. [54] evaluated the pregnancies of transgenic lambs produced by SCNT. They observed that fetal weight, total placenta weight, and mean placentomes weight were greater in pregnancies with live-born lambs, but did not survive compared to pregnancies with live-born lambs that survived. Further deregulation was found in miR-21 and miR-16 in the placenta of non-surviving lambs, causing aberrant expression in their targets.

Cluster four, in light green, contains nine words referring to the epigenetic status of cloned sheep embryos. Morphological evaluation is routinely used as the main parameter of embryo quality. However, due to the limited information provided by its unique evaluation, other parameters have focused on evaluating epigenetics [27,55] and the genetic status of sheep embryos generated by SCNT [9,52].

Finally, cluster five, in purple, includes five words related to the generation of transgenic sheep mediated cloning. Zhang et al. [56] investigated the effect of suppressing the myostatin (MSTN) gene expression in sheep skeletal muscle satellite cell using CRISPR/Cas9 (clustered regularly interspaced short palindromic repeats (CRISPR)/CRISPR-associated protein 9 (Cas9)) technology, to generate lamb clones with better muscle conformation; it has been reported that MSTN gene is responsible for regulating the growth of muscle cells.

It has been 26 years since the first sheep was cloned by applying SCNT, and since then, it has been applied to different domestic and wild species, in some cases with viable newborns. However, SCNT is an inefficient biotechnology, for example, Sheep were one of the first species to be domesticated for newborns lambs have been reported to be from 5.7 to 15% per transferred blastocyst and from 7.1% to 19.5% per segmented embryo [57]. Although sheep are easy to handle and have a relatively short gestation period compared with that of the other species of zootechnical interest [58], their small value and limited potential, do not make them attractive for agricultural use as compared with other livestock species [59]. If we also take into account that the infrastructure used for SCNT is basically the same for all species, nowadays, SCNT applied to sheep reproduction is not profitable. Therefore, the present bibliometric study shows the areas of research in which cloning research in sheep should be directed, which will help those regions where sheep are an important economic and food source.

## 5. Conclusions

Bibliometric studies on SCNT in sheep have not been conducted prior to now. This study collected bibliographic data from 124 papers relating to the application of SCNT in sheep. This amount of information about sheep is smaller than that for other species of zootechnical interest, such as cattle and pigs, although sheep were the first large mammal to be successfully cloned. Since 2001, the number of SCNT-related papers that have been published concerning ovine reproduction has increased and has fluctuated in ensuing years. The authors that have generated more knowledge in this area are (in alphabetical order) Campbell, Hajian, Hosseini, Loi, Nasr-Esfahani, Ptak, and Wilmut. Five research groups were identified, three of which mutually collaborated. The countries with the largest number of published papers were China, Italy, and England. The largest collaboration network among countries comprises China, Iran, Australia, Canada, and United States. The institutions with the highest productivity of SCNT in sheep are the University of Nottingham and the Roslin Institute at the University of Edinburgh. These two institutions are among the top 150 universities in the world. The principal journals where topics about SCNT in sheep are published have an IF ranging from 1.9 to 4.1, whose quartile position is most often in third and fourth place in the JCR thematic categories. These journals are the most suitable for publishing scientific advances in this area. The articles that have been cited more often have addressed topics related to the generation of transgenic animals, recovery of wild species, and xenotransplants. Five main themes were identified in sheep reproduction by SCNT. These themes focused on the competence of in vitro clone embryos, cells used as karyoplasts and their efficiency on embryo development, epigenetic status of clone embryos and their impact on post-implantation development, and generation of transgenic sheep with biomedical and genetic improvement purposes. Concerning the application of SCNT in sheep, these topics are the most relevant, and future studies should focus on solutions to the current challenges in this field of study.

## Figures and Tables

**Table 1:** Bibliometric indicators used to analyze scientific research on the application of SCNT in the production of sheep indexed in the Web of Science.

One-Dimensional Indicators	Multi-Dimensional Indicators
Growth of literature	Cooperation among countries
Most published authors	Cooperation among authors
Most cited papers	Identification of research topics
Country of publication	
Institutions with more published papers	
Publishing journals	

**Table 2:** List of leading authors and the number of citations in the scientific research of sheep production by using SCNT.

Author	Papers	Citations	H-Index	Institution	Research Interests
Campbell	15	4868	34	University of Nottingham	Cloning mammals, transgenic animals, and stem cells
Loi	13	610	19	University of Teramo	Developmental biology, reproductive biotechnologies, nuclear reprogramming, and epigenetic modifications
Ptak	11	595	25	Jagiellonian University	Mechanisms involved in the implantation and placentation of mammals, influence of the environment on the development of organisms
Wilmut	9	4992	69	The University of Edinburgh	Regenerative medicine and cellular reprogramming mechanisms
Hajian	8	139	18	Royan Institute for Biotechnology	Reproductive biotechnologies, mammalian cloning, genetics, and molecular biology
Hosseini	8	139	21	Royan Institute for Biotechnology	Reproductive biotechnologies in wild animals and mammalian cloning
Nasr-Esfahani	8	139	47	Royan Institute for Biotechnology	Reproductive biotechnologies in ruminants
Forouzanfar	7	123	16	Islamic Azad University	Reproductive biotechnologies, cloning of mammals and transgenic animals
Hou	7	57	16	Institute of Crops Sciences	Reproductive biotechnologies and cloning
Lee	7	233	18	Pusan National University	Cloning of mammals
Peura	7	139	20	Genea Biomedx	Reproductive biotechnologies
Walker	7	284	31	South Australian Research & Development Institute	Reproductive biotechnologies and embryonic development in ruminants
Czernik	6	48	15	University of Teramo	Assisted reproduction techniques and reproductive biology
Guan	6	45	13	Guangzhou Institute of Energy Conversion	Reproductive biotechnologies and cloning
Moulavi	6	119	14	Camel Advanced Reproductive Technologies Center	Reproductive biotechnologies in wild animals and mammalian cloning
Choi	5	82	13	University of Nottingham	Developmental biology, genome expression, embryonic development, and mammalian cloning
Iuso	5	41	9	National Institute for Biology	Assisted reproduction techniques and reproductive biology

The author list was ordered based on the number of papers published by each author.

**Table 3:** Main countries with the most published papers concerning the application of SCNT in sheep production.

Country	Number of Published Papers
China	45
Italy	16
England	15
Scotland	15
United States	12
Iran	11
Australia	10
France	6
Poland	6
Canada	5

**Table 4:** Top ten institutions that have contributed published paprs on the application of SCNT in sheep production.

Institution	Country	P	TC	AC	>100	>30	<30	CP	ARWU	QS
1. University of Nottingham	United Kingdom	15	510	34	2	3	10	2	101–150	114
2. The Roslin Institute	United Kingdom	13	1844	141.8	7	3	3	2	-	-
3. Università Degli studi di Teramo	Italy	12	143	11.9	0	3	9	4	-	-
4. China Agricultural University	China	11	79	7.2	0	0	12	5	201–300	591–600
6. Academic Center for Education, Culture & Research (ACECR)	Iran	8	132	16.5	0	2	6	1	-	-
5. Inner Mongolia Agricultural University	China	7	37	5.2	0	0	7	6	-	-
7. Shihezi University	China	6	88	14.6	0	1	5	3	-	-
8. Turretfield Research Centre	Australia	5	244	48.8	1	0	4	0	-	-
9. The University of Edinburgh	United Kingdom	5	253	50.6	1	0	4	3	35	15
10. Northwest A&F University	China	5	50	10	0	2	3	2	401–500	-

P, number of papers; TC, total citations; AC, average number of citations; >100, papers with more than 100 citations; >30, papers between 30 and 99 citations; <30, papers with fewer than 30 citations; CP, contemporary productivity (papers published in the last 10 years); ARWU, Academic Ranking of World Universities; QS, World University Rankings.

**Table 5:** Main journals with greater contributions of SCNT-related articles on sheep.

Journal	Papers	Citations	Impact Factor	JCR Category	Rank, Quartile
*Cellular Reprogramming*	11	155	2.257	Biotechnology and Applied Microbiology	130/156, Q4
				Cell and Tissue Engineering	27/29, Q4
				Genetics and Inheritance	132/175, Q4
*Theriogenology*	10	267	2.923	Reproductive Biology	20/31, Q3
				Veterinary Sciences	21/145, Q1
*Reproduction Fertility and Development*	8	88	1.973	Developmental Biology	33/39, Q4
				Reproductive Biology	27/31, Q4
				Zoology	59/176, Q2
*Molecular Reproduction and Development*	6	110	2.812	Biochemistry and Molecular Biology	230/297, Q4
				Cellular Biology	159/195, Q4
				Developmental Biology	20/39, Q3
				Reproductive Biology	22/31, Q3
*PLoS ONE*	6	98	3.752	Multidisciplinary Sciences	29/74, Q2
*Reproduction in Domestic Animals*	6	42	1.858	Agriculture, Dairy and Animal Science	33/62, Q3
				Reproductive Biology	29/31, Q4
				Veterinary Sciences	55/145, Q2
*Animal Reproduction Sciences*	5	171	2.22	Agriculture, Dairy and Animal Science	22/62, Q2
				Reproductive Biology	23/31, Q3
				Veterinary Sciences	44/145, Q2
*Biology of Reproduction*	5	485	4.161	Reproductive Biology	10/31, Q2

**Table 6:** Most cited articles on SCNT concerning the reproduction of sheep.

Year	Authors	Title	PT	Source	Category	TC
1997	Wilmut I. et al.	Viable offspring derived from fetal and adult mammalian cells [21]	Article	*Nature*	SCNT	3476
1997	Schnieke A. E. et al.	Human factor IX transgenic sheep produced by transfer of nuclei from transfected fetal fibroblasts [25]	Article	*Science*	Transgenic	650
2000	Mccreath K. J. et al.	Production of gene-targeted sheep by nuclear transfer from cultured somatic cells [26]	Article	*Nature*	Transgenic	450
2001	Loi P. et al.	Genetic rescue of an endangered mammal by cross-species nuclear transfer using post-mortem somatic cells [10]	Article	*Nature Biotechnology*	SCNT interspecific	296
2004	Beaujean N. et al.	Effect of limited DNA methylation reprogramming in the normal sheep embryo on somatic cell nuclear transfer [27]	Article	*Biology of Reproduction*	DNA methylation in embryos by SCNT	187
2001	Denning C. et al.	Deletion of the alpha (1,3) galactosyl transferase (ggta1) gene and the prion protein (prp) gene in sheep [28]	Article	*Nature Biotechnology*	Transgenic y Xenotransplants	185
1999	Evans M. J. et al.	Mitochondrial DNA genotypes in nuclear transfer derived cloned sheep [29]	Article	*Nature Genetics*	Heteroplasmy in ovine	169
2001	De Sousa P. A. et al.	Evaluation of gestational deficiencies in cloned sheep fetuses and placentae [30]	Article	*Biology of Reproduction*	Placental insufficiency in clone fetuses	168
2004	Young L. E., Beaujean N.	DNA methylation in the preimplantation embryo: the differing stories of the mouse and sheep [31]	Review Article	*Animal Reproduction Sciences*	Methylation process in clone embryos	121
2003	Young Le. et al.	Conservation of igf2-h19 and igf2r imprinting in sheep: effects of somatic cell nuclear transfer [32]	Article	*Mechanisms of development*	Genomic imprinting in sheep	94
2006	Lee J. H., Campbell K. H. S.	Effects of enucleation and caffeine on maturation promoting factor (mpf) and mitogen activated protein kinase (mapk) activities in ovine oocytes used as recipient cytoplasts for nuclear transfer [33]	Article	*Biology of Reproduction*	Nuclear reprogramming	69
2018	Fan Z. et al.	A sheep model of cystic fibrosis generated by CRISPR/Cas9 disruption of the CFTR gene [34]	Article	*JCI Insight*	Genome Editing	67
2007	Lagutina I. et al.	Comparative aspects of somatic cell nuclear transfer with conventional and zona-free method in cattle, horse, pig and sheep [35]	Article	*Theriogenology*	Modification to conventional SCNT technique	66
2006	Loi P. et al.	Placental abnormalities associated with post-natal mortality in sheep somatic cell clones [2]	Article	*Theriogenology*	Placental abnormalities	57
2008	Palmieri C. et al.	A review of the pathology of abnormal placentae of somatic cell nuclear transfer clone pregnancies in cattle, sheep and mice [36]	Review Article	*Veterinary Pathology*	Placental abnormalities	56
2007	Bowles E. J. et al.	Contrasting effects of in vitro fertilization and nuclear transfer on the expression of mtDNA replication factors [37]	Article	*Genetics*	Mitochondrial DNA replication factors	47
2006	Alexander B. et al.	The effects of 6-dimethylaminopurine (6-DMAP) and cycloheximide (CHX) on the development and chromosomal complement of sheep parthenogenetic and nuclear transfer embryos [38]	Article	*Molecular Reproduction and Development*	Activation of cario-cytoplasmic complexes	46

PT, publication type; TC, total citations.

**Table 7:** Main words used most frequently in published paprs concerning the application of SCNT in sheep.

Keywords	Frequency	Keywords	Frequency
SCNT	90	Mice	19
Sheep	56	Oocyte	18
Gene Expression	36	Somatic Cell	16
Embryo	32	Transgenic	16
In Vitro	28	Oocyte Activation	15
Fetal	22	Fetal Fibroblasts	14
Cattle	20	Bovine Embryo	13

## Data Availability

Data are available from first author José Roberto Vazquez-Avendaño (robertmizer@gmail.com) upon request.
